# Burden of illness in US hospitals due to carbapenem-resistant Gram-negative urinary tract infections in patients with or without bacteraemia

**DOI:** 10.1186/s12879-021-06229-x

**Published:** 2021-06-14

**Authors:** Ryan K. Shields, Yun Zhou, Hemanth Kanakamedala, Bin Cai

**Affiliations:** 1 University of Pittsburgh, Pittsburgh, PA USA; 2 Genesis Research Inc., Hoboken, NJ USA; 3 Shionogi Inc., 300 Campus Drive, Suite 100, Florham Park, NJ 07932 USA

**Keywords:** Bacteraemia, Carbapenem resistance, Healthcare burden, Urinary tract infection

## Abstract

**Background:**

Urinary tract infections (UTIs) are the most common infections caused by Gram-negative bacteria and represent a major healthcare burden. Carbapenem-resistant (CR) strains of Enterobacterales and non-lactose fermenting pathogens further complicate treatment approaches.

**Methods:**

We conducted a retrospective analysis of the US Premier Healthcare Database (2014–2019) in hospitalised adults with a UTI to estimate the healthcare burden of Gram-negative CR UTIs among patients with or without concurrent bacteraemia.

**Results:**

Among the 47,496 patients with UTI analysed, CR infections were present in 2076 (4.4%). Bacteraemia was present in 24.5% of all UTI patients, and 1.7% of these were caused by a CR pathogen. The most frequent CR pathogens were *Pseudomonas aeruginosa* (49.4%) and *Klebsiella pneumoniae* (14.2%). Patients with CR infections had a significantly longer hospital length of stay (LOS) (median [range] 8 [5–12] days vs 6 [4–10] days, *P* < 0.001), were less likely to be discharged home (38.4% vs 51.0%, *P* < 0.001), had a higher readmission rate (22.6% vs 13.5%, *P* < 0.001), and had greater LOS-associated charges (mean US$ 91,752 vs US$ 66,011, *P* < 0.001) than patients with carbapenem-susceptible (CS) infections, respectively. The impact of CR pathogens was greater in patients with bacteraemia (or urosepsis) and these CR urosepsis patients had a significantly higher rate of mortality than those with CS urosepsis (10.5% vs 6.0%, *P* < 0.001).

**Conclusions:**

Among hospitalised patients with UTIs, the presence of a CR organism and bacteraemia increased the burden of disease, with worse outcomes and higher hospitalisation charges than disease associated with CS pathogens and those without bacteraemia.

**Supplementary Information:**

The online version contains supplementary material available at 10.1186/s12879-021-06229-x.

## Background

Urinary tract infections (UTIs) are the most frequent infections caused by Gram-negative bacteria [1–3]. UTIs represent a considerable healthcare burden that manifests in emergency department visits and/or acute care hospitalisations that are associated with significant healthcare costs [1–3]. Between 1998 and 2011, an increasing number of hospitalisations due to UTI were recorded in the USA, particularly among women and older patients [4]. At least 10–15% of UTIs in patients visiting emergency departments are associated with urosepsis [5], which may account for up to 25% of all sepsis cases and is associated with an increased risk of mortality [1, 6–8].

The most common urinary pathogens are Enterobacterales [8, 9] but non-lactose fermenting bacteria are also increasingly identified as causative pathogens [9, 10].

Carbapenem resistance is a well-recognised clinical problem and carbapenem-resistant (CR) Gram-negative pathogens are a global public health threat [11, 12]. UTIs are among the top sources of CR infections [10, 13–17]. Although CR Enterobacterales (CRE), including *Escherichia coli* and *Klebsiella pneumoniae*, have received considerable attention for their impact on morbidity and mortality, it is also important to note that CR non-lactose fermenting bacteria, such as CR *Acinetobacter baumannii* and CR *Pseudomonas aeruginosa* are common in many regions and known to negatively impact clinical outcomes [13, 18–20]. The prevalence of CR pathogens remains higher in non-fermenters than in fermenters in the USA and in other regions [13, 21, 22]. While new antibiotics have been approved for UTIs caused by Enterobacterales, treatment options remain limited for those infections caused by CR non-fermenting bacteria [11].

The objective of this retrospective study was to describe the total healthcare burden of UTIs caused by Gram-negative CR pathogens in hospitalised patients, with or without bacteraemia secondary to UTI.

## Methods

### Study design

This was a retrospective analysis of data from the Premier Healthcare Database, which collects anonymised patient-level data from over 700 US hospitals annually. The analysis is based on data from the 315 hospitals that provided microbiology test results, including specimen site, pathogen, and antibiotic susceptibility results for hospitalised patients between January 1, 2014 and June 30, 2019.

Adult patients (≥18 years old) with at least one hospital admission with a UTI caused by Gram-negative pathogen(s) and carbapenem susceptibility results were eligible for inclusion. UTIs were defined by a positive urine culture due to a Gram-negative organism and receipt of antibiotics with activity against Gram-negative pathogens between 2 days before and 3 days after an index urine culture. Patients with a urine culture collected before admission or after discharge were excluded from the analysis. Bacteraemia or urosepsis, which was diagnosed on or after the index UTI and during the same hospital admission, was defined as a positive blood culture due to the same pathogen detected in the index urine culture (Supplementary Figure 1). ICD-9 (038.x, 995.91) and ICD-10 (A40.x and A41.x) codes for sepsis were used to exclude patients who had a diagnosis of sepsis or bloodstream infection for any pathogen at any time prior to diagnosis of UTI for both bacteraemic and non-bacteraemic cohorts.

Carbapenem susceptibility and resistance were defined according to Clinical and Laboratory Standards Institute breakpoints to doripenem, ertapenem, imipenem, and meropenem, which have been updated previously in 2012 for Enterobacterales and *P. aeruginosa*, and in 2015 for *A. baumannii* [23]. For analysis, carbapenem resistance was defined as resistance or intermediate susceptibility to any carbapenem tested, and included all *Stenotrophomonas maltophilia*. Patients with multiple pathogens were considered to have CR infections if any of the uropathogens were CR during their hospitalisation. For patients with multiple positive cultures yielding the same pathogen, the first CR result was selected as the index culture for CR patients and the first positive culture was selected as the index culture for carbapenem-susceptible (CS) patients. The collection date of the index culture was defined as the index date. For patients with multiple hospitalisations for either UTI or other diseases, the first hospitalisation meeting the index culture definition was the index hospitalisation.

### Outcomes

Patient characteristics, including demographics (age, sex, and race), comorbidities, admission source, admission time, and time of infection onset, were stratified by CR and bacteraemia status. Comparative outcomes, including in-hospital mortality, day 14 and day 30 all-cause mortality rates from the index culture, overall hospital length of stay (LOS), infection-associated LOS, intensive care unit (ICU) LOS, infection-associated ICU LOS, total hospitalisation charges (charge is the amount that the hospital charged to insurance companies, which was applicable to the duration of hospitalisation or ICU stay or infection-associated ICU stay), and discharge status, were compared by CR and bacteraemia status.

LOS was defined as the number of days from admission to discharge. Infection-associated LOS was defined as the number of days from index urine culture date to discharge and calculated as LOS − index culture day + 1. ICU LOS was defined as the number of days from the first to the last ICU service day. Infection-associated ICU LOS was defined as the number of days from the first to the last ICU service day depending on the timing of the positive culture: if a positive culture was obtained within 2 days of ICU admission, the entire ICU LOS was used; if the culture was obtained during ICU stay, the LOS was taken as the number of days from the ICU index culture to discharge from the ICU. Patients with more than one inpatient stay for the same pathogen that caused the index UTI after index hospitalisation were flagged as readmission and the time to readmission was calculated as the number of days from discharge of the index hospitalisation to the first readmission date.

### Statistical analyses

Data are presented using descriptive statistics, using number (%) for categorical variables (age group, sex, pathogen type, baseline comorbidities, baseline Charlson Comorbidity Index [CCI] categories, origin of admission, type of admission, ICU admission, ICU at index culture, discharge status, readmission) and using mean (standard deviation [SD]) and median (interquartile range [Q1–Q3]) for continuous variables (age, baseline CCI score, time between admission and index culture, overall LOS, infection-associated LOS, ICU LOS, infection-associated ICU LOS, time between discharge to readmission, total LOS charges, and infection-associated ICU-LOS charges).

A univariate comparison between CR and CS patients was conducted using a chi-squared test for categorical variables, a Student’s *t* test for mean values, and a Wilcoxon rank sum test for median values, and included the following variables: demographics (age, sex), origin of admission, type of admission, comorbidities, CCI, Gram-negative pathogens (*A. baumannii*, *E. coli*, *Enterobacter aerogenes*, *Enterobacter cloacae*, *Klebsiella oxytoca*, *K. pneumoniae*, *Morganella morganii*, *P. aeruginosa*, *Proteus mirabilis*, *Serratia marcescens*, and *S. maltophilia*), discharge status, readmission, ICU admission, LOS (total and infection-associated), ICU LOS (total and infection-associated), and days between admission and index culture. Significant variables (*P* < 0.1) from univariate analyses were included in the multivariable logistic regression to assess the risk of developing bacteraemia from UTI.

## Results

### Patient demographics and clinical characteristics

A total of 47,496 hospitalised patients with UTI were included. Overall, 24.5% (*n* = 11,629) had UTI complicated with bacteraemia and 75.5% (*n* = 35,867) had UTI without bacteraemia (Supplementary Figure 1). Altogether, 4.4% of patients had CR infections (*n* = 2076): 1.7% (201/11629) with UTI and bacteraemia, and 5.2% (1875/35867) with UTI without bacteraemia. During the study period, a gradual increase was observed in the proportion of patients with CR UTIs from 4.1% in 2014 to 5.5% in 2018.

The mean age of patients was 69 years with no significant difference between patients with CR or CS UTI (Table 1); there were nearly twice as many females (*n* = 31,447) as males (*n* = 16,049) overall. Among patients with CR pathogens, there was a higher percentage of males than females, especially among those with bacteraemia (61.7% vs 38.3%) (Table 1). Most patients were admitted through an emergency department (83.9%, *n* = 39,891) and had a non-healthcare facility origin of their infection (76.3%, *n* = 36,231) (Table 1).

**Table 1:** Characteristics for hospitalised patients with Gram-negative UTIs according to presence of bacteraemia and carbapenem resistance

Characteristic	Overall, ***N*** = 47,496			With bacteraemia, ***n*** = 11,629			Without bacteraemia, ***n*** = 35,867		
	CR, ***n*** = 2076	CS, ***n*** = 45,420	***P*** value	CR, ***n*** = 201	CS, ***n*** = 11,428	***P*** value	CR, ***n*** = 1875	CS, ***n*** = 33,992	***P*** value
Age, years									
*Mean (SD)*	69.1 (15.36)	68.9 (16.7)	0.563	68.6 (14.88)	68.34 (15.88)	0.816	69.15 (15.41)	69.09 (16.91)	0.859
*Median (Q1–Q3)*	71 (61.0–81.0)	72 (60.0–82.0)	0.335	70 (63.0–80.0)	70 (59.0–81.0)	0.962	71 (60.0–81.0)	72 (60.0–83.0)	0.111
*Minimum, Maximum*	18, 89	18, 89		19, 89	18, 89		18, 89	18, 89	
Sex, n (%)									
*Female*	968 (46.6)	30,479 (67.1)	< 0.001	77 (38.3)	6679 (58.4)	< 0.001	891 (47.5)	23,800 (70.0)	< 0.001
*Male*	1108 (53.4)	14,941 (32.9)		124 (61.7)	4749 (41.6)		984 (52.5)	10,192 (30.0)	
Race, n (%)									
*White*	1597 (76.9)	33,861 (74.6)	< 0.001	155 (77.1)	8190 (71.7)	0.073	1442 (76.9)	25,671 (75.5)	0.004
*Black*	319 (15.4)	6570 (14.5)		31 (15.4)	1706 (14.9)		288 (15.4)	4864 (14.3)	
*Other*	151 (7.3)	4527 (10.0)		15 (7.5)	1388 (12.1)		136 (7.3)	3139 (9.2)	
*Unable to determine*	9 (0.4)	462 (1.0)		0 (0.0)	144 (1.3)		9 (0.5)	318 (0.9)	
Admission source, n (%)									
*Non-healthcare facility point of origin*	1481 (71.3)	34,750 (76.5)	< 0.001	136 (67.7)	9133 (79.9)	< 0.001	1345 (71.7)	25,617 (75.4)	0.002
*Transfer from other facility/hospital*	214 (10.3)	3970 (8.7)		19 (9.5)	720 (6.3)		195 (10.4)	3250 (9.6)	
*Transfer from SNF/ intermediate care facility (clinic)*	308 (14.8)	5499 (12.1)		37 (18.4)	1217 (10.7)		271 (14.5)	4282 (12.6)	
*Other/unavailable*	73 (3.5)	1201 (2.6)		9 (4.5)	358 (3.1)		64 (3.4)	843 (2.5)	
Admission type, n (%)									
*Elective*	154 (7.4)	2844 (6.3)	< 0.001	13 (6.5)	379 (3.3)	0.015	141 (7.5)	2465 (7.3)	0.022
*Emergency*	1664 (80.2)	38,227 (84.2)		166 (82.6)	10,233 (89.5)		1498 (79.9)	27,994 (82.4)	
*Trauma centre*	8 (0.4)	191 (0.4)		0 (0.0)	17 (0.2)		8 (0.4)	174 (0.5)	
*Urgent*	245 (11.8)	4042 (8.9)		22 (10.9)	781 (6.8)		223 (11.9)	3261 (9.6)	
*Information not available*	5 (0.2)	116 (0.3)		0 (0.0)	18 (0.2)		5 (0.3)	98 (0.3)	
Baseline CCI Score									
*Mean (SD)*	3.5 (2.6)	2.9 (2.5)	< 0.001	3.3 (2.7)	2.7 (2.5)	0.002	4.0 (2.6)	3.0 (2.5)	< 0.001
*Median (Q1–Q3)*	3 (2.0–5.0)	2 (1.0–4.0)	< 0.001	3 (2.0–5.0)	2 (1.0–4.0)	0.001	3 (2.0–5.0)	2 (1.0–4.0)	< 0.001
*Minimum, Maximum*	0, 15	0, 18		0, 13	0, 17		0, 15	0, 18	
Total number of patients with at least one comorbidity, n (%)	1874 (90)	38,176 (84)	< 0.001	174 (87)	9378 (82)	0.098	1700 (91)	28,798 (85)	< 0.001
Baseline Charlson comorbidities, n (%)									
*Renal disease*	783 (37.7)	13,423 (29.6)	< 0.001	76 (37.8)	3387 (29.6)	0.012	707 (37.7)	10,036 (29.5)	< 0.001
*Chronic pulmonary disease*	680 (32.8)	12,712 (28.0)	< 0.001	47 (23.4)	2554 (22.4)	0.727	633 (33.8)	10,158 (29.9)	< 0.001
*Congestive heart failure*	651 (31.4)	12,161 (26.8)	< 0.001	48 (23.9)	2544 (22.3)	0.584	603 (32.2)	9617 (28.3)	< 0.001
*Peripheral vascular disease*	277 (13.3)	4680 (10.3)	< 0.001	18 (9.0)	911 (8.0)	0.610	259 (13.8)	3769 (11.1)	< 0.001
*Cerebrovascular disease*	248 (12.0)	4771 (10.5)	0.037	21 (10.5)	847 (7.4)	0.104	227 (12.1)	3924 (11.5)	0.458
*Myocardial infarction*	222 (10.7)	5284 (11.6)	0.191	21 (10.5)	1392 (12.2)	0.456	201 (10.7)	3892 (11.4)	0.333
*Diabetes without chronic complication*	681 (32.8)	15,043 (33.1)	0.765	62 (30.9)	3913 (34.2)	0.314	619 (33.0)	11,130 (32.7)	0.808
*Diabetes with chronic complication*	289 (13.9)	4996 (11.0)	< 0.001	21 (10.5)	1217 (10.7)	0.927	268 (14.3)	3779 (11.1)	< 0.001
*Mild liver disease*	121 (5.8)	2916 (6.4)	0.281	10 (5.0)	870 (7.6)	0.161	111 (5.9)	2046 (6.0)	0.861
*Moderate or severe liver disease*	52 (2.5)	930 (2.1)	0.152	7 (3.5)	240 (2.1)	0.178	45 (2.4)	690 (2.0)	0.271
*Dementia*	272 (13.1)	5333 (11.7)	0.060	25 (12.4)	1205 (10.5)	0.387	247 (13.2)	4128 (12.1)	0.185
*Hemiplegia or paraplegia*	330 (15.9)	2469 (5.4)	< 0.001	29 (14.4)	413 (3.6)	0.000	301 (16.1)	2056 (6.1)	< 0.001
*Rheumatic disease*	85 (4.1)	1987 (4.4)	0.541	8 (4.0)	489 (4.3)	0.835	77 (4.1)	1498 (4.4)	0.537
*Peptic ulcer disease*	33 (1.6)	793 (1.8)	0.594	3 (1.5)	156 (1.4)	0.877	30 (1.6)	637 (1.9)	0.393
*Any malignancy*	209 (10.1)	4548 (10.0)	0.936	23 (11.4)	1170 (10.2)	0.577	186 (9.9)	3378 (9.9)	0.980
*Metastatic solid tumour*	111 (5.4)	1967 (4.3)	0.027	11 (5.5)	484 (4.2)	0.389	100 (5.3)	1483 (4.4)	0.046
*AIDS/HIV*	6 (0.3)	158 (0.4)	0.655	2 (1.0)	49 (0.4)	0.228	4 (0.2)	109 (0.3)	0.419

*AIDS* Acquired immune deficiency syndrome, *CCI* Charlson Comorbidity Index, *CR* Carbapenem resistant, *CS* Carbapenem susceptible, *HIV* Human immune-deficiency virus, *SD* Standard deviation, *SNF* Skilled nursing facility, *UTI* Urinary tract infection

Overall, 84.3% of patients had at least one comorbidity. Patients with CR pathogens had significantly higher mean baseline CCI than those with CS pathogens (3.5 vs 2.9, *P* < 0.001) (Table 1). Comorbidities that were more common among patients with CR versus CS UTIs were congestive heart failure (31.4% vs 26.8%, respectively, *P* < 0.001), chronic pulmonary disease (32.8% vs 28.0%, respectively, *P* < 0.001), diabetes without chronic complications (32.8% vs 33.1%, respectively), and renal disease (37.7% vs 29.6%, respectively, *P* < 0.001); patients with CR were also more likely to have peripheral vascular disease, diabetes with chronic complications, and hemiplegia/paraplegia (*P* < 0.001 in each case).

### Distribution of baseline pathogens

**Fig. 1:**
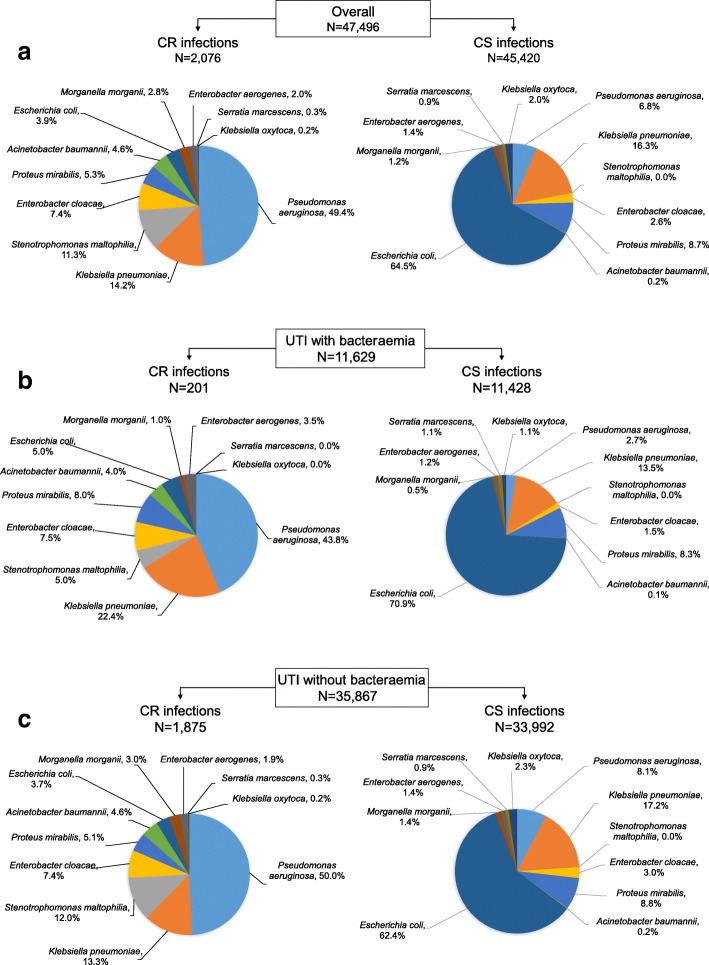
Distribution of baseline pathogens in hospitalised patients with Gram-negative UTIs, according to presence of bacteraemia and carbapenem resistance. **a** Overall. **b** UTI with bacteraemia. **c** UTI without bacteraemia. *CR* Carbapenem resistant, *CS* Carbapenem susceptible, *UTI* Urinary tract infection

The most frequent causative pathogen overall was *E. coli* (61.9%), followed by *K. pneumoniae* (16.2%), and *P. aeruginosa* (8.6%). The most frequent CR organism isolated from UTI patients was *P. aeruginosa* (49.4%), followed by *K. pneumoniae* (14.2%) and *S. maltophilia* (11.3%) (Fig. 1). The majority of CR pathogens in UTI patients with bacteraemia were *P. aeruginosa* (43.8%) and *K. pneumoniae* (22.4%). Although *E.coli* was the most frequent uropathogen, most isolates were susceptible to carbapenems and prevalence of CR *E. coli* was low (overall 3.9%; bacteraemia 5.0%; non-bacteraemia 3.7%)*.* Of 181 hospitals contributing cases to the analysis, 63.5% had < 10 CR UTI patients and 4.4% had ≥50 CR UTI patients during the study period.

### Outcomes

Mean time to identification of CR pathogens (defined as day of admission to day of culture) was significantly longer than that of CS pathogens (overall: 3.6 days vs 2.1 days; UTI patients with bacteraemia: 5.1 days vs 1.5 days; *P* < 0.001 for both) (Table 2). A higher proportion of CS pathogens (82.3%) than CR pathogens (69.2%) were identified on the day of the admission among patients with bacteraemia (Table 2).

**Table 2:** Time to UTIs and lengths of stay according to presence of bacteraemia and carbapenem resistance

Characteristic	Overall, ***N*** = 47,496			With bacteraemia, ***n*** = 11,629			Without bacteraemia, ***n*** = 35,867		
	CR, ***n*** = 2076	CS, ***n*** = 45,420	***P*** value	CR, ***n*** = 201	CS, ***n*** = 11,428	***P*** value	CR, ***n*** = 1875	CS, ***n*** = 33,992	***P*** value
Time between admission and index culture, days									
*Mean (SD)*	3.6 (10.9)	2.1 (9.3)	< 0.001	5.1 (13.8)	1.5 (3.9)	< 0.001	3.5 (10.5)	2.3 (10.5)	< 0.001
*Median (Q1–Q3)*	1 (1.0–2.0)	1 (1.0–1.0)	< 0.001	1 (1.0–1.0)	1 (1.0–1.0)	< 0.001	1 (1.0–2.0)	1 (1.0–1.0)	< 0.001
*1 day prior to admission, n (%)*	186 (9.0)	4285 (9.4)	< 0.001	23 (11.4)	1256 (11.0)	< 0.001	163 (8.7)	3029 (8.9)	< 0.001
*Same day as admission, n (%)*	1300 (62.6)	31,927 (70.3)		139 (69.2)	9409 (82.3)		1161 (61.9)	22,518 (66.2)	
Overall LOS^a^, days									
*Mean (SD)*	12.5 (23.8)	9.3 (23.0)	< 0.001	18.0 (25.0)	9.1 (11.8)	< 0.001	11.9 (23.6)	9.4 (25.7)	< 0.001
*Median (Q1–Q3)*	8 (5.0–12.0)	6 (4.0–10.0)	< 0.001	9 (6.0–16.0)	7 (5.0–10.0)	< 0.001	8 (5.0–12.0)	6 (4.0–10.0)	< 0.001
*1–2, n (%)*	57 (2.7)	1683 (3.7)	< 0.001	5 (2.5)	340 (3.0)	< 0.001	52 (2.8)	1343 (4.0)	< 0.001
*3–6, n (%)*	729 (35.1)	21,109 (46.5)		47 (23.4)	5256 (46.0)		682 (36.4)	15,853 (46.6)	
*7–10, n (%)*	622 (30.0)	11,922 (26.2)		66 (32.8)	3305 (28.9)		556 (29.7)	8617 (25.4)	
*11–15, n (%)*	303 (14.6)	5313 (11.7)		32 (15.9)	1355 (11.9)		271 (14.5)	3958 (11.6)	
*16+, n (%)*	365 (17.6)	5393 (11.9)		51 (25.4)	1172 (10.3)		314 (16.7)	4221 (12.4)	
Infection-associated LOS^b^, days									
*Mean (SD)*	9.9 (16.3)	8.2 (15.3)	< 0.001	13.9 (16.1)	8.6 (9.9)	< 0.001	9.5 (16.3)	8.1 (16.7)	< 0.001
*Median (Q1–Q3)*	7 (5.0–11.0)	6 (4.0–9.0)	< 0.001	9 (6.0–14.0)	7 (5.0–10.0)	< 0.001	7 (5.0–10.0)	6 (4.0–9.0)	< 0.001
*1–2, n (%)*	87 (4.2)	1669 (3.7)	< 0.001	6 (3.0)	314 (2.7)	< 0.001	81 (4.3)	1355 (4.0)	< 0.001
*3–6, n (%)*	812 (39.1)	22,924 (50.5)		45 (22.4)	5327 (46.6)		767 (40.9)	17,597 (51.8)	
*7–10, n (%)*	639 (30.8)	12,125 (26.7)		72 (35.8)	3435 (30.1)		567 (30.2)	8690 (25.6)	
*11–15, n (%)*	299 (14.4)	4951 (10.9)		36 (17.9)	1349 (11.8)		263 (14.0)	3602 (10.6)	
*16+, n (%)*	239 (11.5)	3751 (8.3)		42 (20.9)	1003 (8.8)		197 (10.5)	2748 (8.1)	
ICU occurred during index hospitalisation, n (%)	670 (32.3)	15,737 (34.7)	0.026	114 (56.7)	5156 (45.1)	0.001	556 (29.7)	10,581 (31.1)	0.179
ICU LOS, days									
*Mean (SD)*	9.0 (21.8)	5.8 (17.7)	< 0.001	14.4 (28.0)	5.2 (12.0)	0.001	7.9 (20.1)	6.0 (19.9)	0.034
*Median (Q1–Q3)*	3 (1.0–8.0)	3 (1.0–6.0)	0.100	3 (1.0–10.0)	3 (1.0–5.0)	0.172	3 (1.0–7.5)	3 (1.0–7.0)	0.405
Infection-associated ICU stay, n (%)	520 (25.1)	12,940 (28.5)	0.001	100 (49.8)	4713 (41.2)	0.015	420 (22.4)	8227 (24.2)	0.076
ICU at index culture, n (%)	413 (19.9)	9874 (21.7)	0.046	76 (37.8)	3587 (31.4)	0.052	337 (18.0)	6287 (18.5)	0.571
Infection-associated ICU LOS^c^, days									
*Mean (SD)*	6.5 (14.6)	4.9 (10.1)	0.013	9.2 (17)	4.8 (10.2)	0.011	5.9 (13.9)	5.0 (10.1)	0.195
*Median (Q1–Q3)*	3 (1.0–7.0)	3 (1.0–6.0)	0.173	3 (1.0–8.5)	3 (1.0–5.0)	0.373	3 (1.0–6.0)	3 (1.0–6.0)	0.381

Patients with index culture taken before the admission or after discharge were excluded from the analyses

*CR* Carbapenem resistant, *CS* Carbapenem susceptible, *ICU* Intensive care unit, *LOS* Length of stay, *SD* Standard deviation, *UTI* Urinary tract infection

^a^Overall LOS: number of days from admission to discharge

^b^Infection-associated LOS: number of days from index culture date to discharge

^c^Infection-associated ICU LOS: if a positive culture was obtained within 2 days of ICU admission, the entire ICU LOS was used; if the culture was obtained during ICU stay, the LOS was taken as the number of days from the index culture to discharge from the ICU

Both the mean and median overall hospital LOS were significantly (*P* < 0.001) longer in patients with CR compared with CS pathogens (Table 2); this was true overall and among patients with or without bacteraemia. Findings were similar for infection-associated LOS (Table 2). In patients with bacteraemia, those with a CR pathogen were more likely to have been admitted to the ICU related to an infection than those with a CS pathogen during index hospitalisation (49.8% vs 41.2%, *P* = 0.015); the mean infection-associated ICU LOS was twice as long in patients with a CR pathogen than in those with a CS pathogen (9.2 days vs 4.8 days, *P* = 0.011) (Table 2). No significant difference in rate of ICU admission or ICU LOS was identified between CR and CS UTIs among patients without bacteraemia (Table 2).

Overall, patients with CR infections were less likely to be discharged home (38.4% vs 51.0%) and more likely to be transferred to another institution, mainly a skilled nursing facility, another acute-care facility, long-term care or rehabilitation facility (42.2% vs 33.0%, *P* < 0.001) than those with CS infection (Table 3). Crude in-hospital mortality among patients with UTI complicated with bacteraemia was significantly higher for those with CR compared with CS infections (10.5% vs 6.0%, *P* < 0.001) (Table 3). There was no difference in death rates within 14 days and within 30 days of index UTI culture for inpatient mortality between CR and CS infections in patients with or without bacteraemia (Supplementary Table 1). In both groups, UTI with bacteraemia and UTI without bacteraemia, CR infections tended to have a higher mortality rate than CS infections, with few exceptions, though sample sizes were small for some pathogens (Supplementary Table 2).

**Table 3:** Hospital disposition and re-admissions according to presence of bacteraemia and carbapenem resistance

	Overall, ***N***=47,496			With bacteraemia, ***n***=11,629			Without bacteraemia, ***n***=35,867		
**Characteristic**	**CR,** ***n*=2076**	**CS,** ***n*=45,420**	***P*** **value**	**CR,** ***n*=201**	**CS,** ***n*=11,428**	***P*** **value**	**CR,** ***n*=1875**	**CS,** ***n*=33,992**	***P*** **value**
Discharge status, n (%)									
*In-hospital death*	96 (4.6)	2100 (4.6)	<0.001	21 (10.5)	690 (6.0)	<0.001	75 (4.0)	1410 (4.1)	<0.001
*Home*	798 (38.4)	23,174 (51.0)		62 (30.8)	6321 (55.3)		736 (39.3)	16,853 (49.6)	
*Hospice*	108 (5.2)	2049 (4.5)		14 (7.0)	463 (4.1)		94 (5.0)	1586 (4.7)	
*Other*	197 (9.5)	3097 (6.8)		18 (9.0)	655 (5.7)		179 (9.5)	2442 (7.2)	
*Transfer*	877 (42.2)	15,000 (33.0)		86 (42.8)	3299 (28.9)		791 (42.2)	11,701 (34.4)	
Patients who were alive when they were discharged from hospital, n (%)	CR, *n*=1980	CS, *n*=43,320	*P* value	CR, *n*=180	CS, *n*=10,738	*P* value	CR, *n*=1800	CS, *n*=32,582	*P* value
Readmission with the same pathogen^a^	448 (22.6)	5845 (13.5)	<0.001	35 (19.4)	1314 (12.2)	0.004	413 (22.9)	4531 (13.9)	<0.001
*Readmission within 30 days*	136 (6.9)	1174 (2.7)		12 (6.7)	263 (2.5)		124 (6.9)	911 (2.8)	
*Readmission between 31–60 days*	60 (3.0)	802 (1.9)		9 (5.0)	204 (1.9)		51 (2.8)	598 (1.8)	
*Readmission between 61–90 days*	49 (2.5)	547 (1.3)		3 (1.7)	121 (1.1)		46 (2.6)	426 (1.3)	
*Readmission beyond 90 days*	203 (10.3)	3322 (7.7)		11 (6.1)	726 (6.8)		192 (10.7)	2596 (8.0)	
Days between discharge from index hospitalisation to readmission in patients who were alive when they were discharged from hospital									
*Mean (SD)*	168.1 (234.8)	244.4 (295.2)	<0.001	102.4 (157.2)	251.6 (304.9)	<0.001	173.7 (239.6)	242.3 (292.3)	<0.001
*Median (Q1–Q3)*	81 (25.0–201.5)	121 (39.0–341.0)	<0.001	52 (19.0–121.0)	117 (38.0–356.0)	<0.001	83 (27.0–210.0)	122 (39.0–338.0)	<0.001

*CR* Carbapenem resistant, *CS* Carbapenem susceptible, *SD* Standard deviation, *UTI* Urinary tract infection

^a^Readmission: patients with > 1 inpatient encounter were flagged as readmission. Time to readmission was calculated as the number of days from discharge of the index hospitalisation to the first readmission date

Readmission rates were higher among patients with CR UTI compared with a CS UTI (22.6% vs 13.5%, *P* < 0.001); this was true for patients with or without bacteraemia (Table 3). Within 30 days, more patients with CR infections than CS infections were readmitted to the same hospital (Table 3), and readmission occurred earlier for patients with CR pathogens than those with CS pathogens. Among those with CR pathogens, median time to readmission was 52 days in patients with bacteraemia and 83 days in patients without bacteraemia (Table 3).

The mean LOS-associated charges were higher in patients with CR pathogens compared with CS pathogens, particularly those with bacteraemia, for which charges were nearly three-times higher than those for patients with CS pathogens (US$ 178,176 vs US$ 64,838, *P* < 0.001) (Supplementary Table 3). Similar findings were seen for infection-associated ICU LOS-associated charges and infection-associated ICU LOS charges (Supplementary Table 3).

### Risk factors for UTI with bacteraemia

Risk factors for developing bacteraemia from a UTI were analysed first in univariate analysis (Supplementary Table 4), and subsequently in multivariate analysis (Table 4). Bacteraemia was more likely to occur in patients with a CS compared with a CR pathogen (odds ratio [OR] 1.75, *P* < 0.0001) and was twice as likely to develop in males compared with females (OR 2.00, *P* < 0.0001) and in those admitted to the ICU (OR 2.14, *P* < 0.0001). Additional variables associated with a significantly increased likelihood of bacteraemia were non-white race, the presence of urinary catheters, patients who underwent urinary surgery, and being infected by *E. coli* or *K. pneumoniae*, while being infected by non-lactose fermenting pathogens was associated with a significantly lower likelihood of the presence of bacteraemia (Table 4).

**Table 4:** Risk factors associated with the development of bacteraemia in patients hospitalised with UTIs

Factor	Adjusted Odds Ratio (95% CI)	***P*** value
Age, 46–65 vs 18–45 years	1.199 (1.104 to 1.302)	< 0.0001
Age, > 65 vs 18–45 years	1.034 (0.957 to 1.116)	0.3961
Male vs Female	1.999 (1.907 to 2.095)	< 0.0001
Non-White vs White	1.226 (1.166 to 1.289)	< 0.0001
Admitted to ICU vs No	2.142 (2.036 to 2.253)	< 0.0001
Had urine catheters vs No	1.237 (1.164 to 1.314)	< 0.0001
Had urinary surgery vs No	1.428 (1.333 to 1.529)	< 0.0001
*Escherichia coli* vs Other	2.026 (1.9 to 2.159)	< 0.0001
*Klebsiella pneumoniae* vs Other	1.447 (1.333 to 1.571)	< 0.0001
*Acinetobacter baumannii* vs Other	0.587 (0.342 to 1.005)	0.0524
*Pseudomonas aeruginosa* vs Other	0.628 (0.554 to 0.712)	< 0.0001
*Stenotrophomonas maltophilia* vs Other	0.349 (0.18 to 0.675)	0.0018
CS vs CR	1.754 (1.484 to 2.073)	< 0.0001
Onset HA (after 3 days) vs CA (within 3 days)	0.229 (0.209 to 0.252)	< 0.0001

*CA* Community acquired, *CR* Carbapenem resistant, *CS* Carbapenem susceptible, *HA* Hospital acquired, *ICU* Intensive care unit, *UTI* Urinary tract infection

## Discussion

The results of the current analysis of UTI patients from the Premier Healthcare Database, hospitalised between 2014 and 2019 with or without bacteraemia, suggest that the presence of a CR organism increases the burden of disease and results in worse outcomes, such as increased in-hospital mortality, prolonged hospital and ICU stays, as well as increased charges associated with hospitalisation. Patients with CR UTIs were more likely to have multiple comorbidities, as suggested by the significantly higher CCI score (3.5 vs 2.9, *P* < 0.001). When UTIs were complicated by bacteraemia in the presence of CR pathogens, adverse outcomes were more problematic.

Carbapenem resistance is a global problem in both non-lactose fermenters and Enterobacterales [13, 21]. Prevalence of CR pathogens remains higher in non-fermenters than in fermenters in the US and in other regions [13, 21, 22]. In the current analysis, non-fermenters were the more frequent cause of CR UTI. Enterobacterales were found in the majority of CS UTIs and were associated with a greater likelihood of developing bacteraemia. In contrast, the presence of non-fermenters was associated with a lower risk of bacteraemia.

Recently, several new antibiotic agents have been approved for the treatment of complicated UTIs (cUTIs), including β-lactam/β-lactamase inhibitor combinations such as ceftazidime-avibactam, ceftolozane-tazobactam, meropenem-vaborbactam, imipenem-cilastatin-relebactam, and plazomicin, a novel aminoglycoside, and cefiderocol, a novel siderophore cephalosporin [24]. These agents have been shown to be efficacious in the treatment of cUTIs caused by Enterobacterales or *P. aeruginosa*, as demonstrated in large, randomised, double-blind, prospective clinical studies [24]. Furthermore, the efficacy of some of the abovementioned agents against CRE, in a variety of infections, has been confirmed in small pathogen-focused studies, in which colistin or best-available therapy was the control arm [24, 25]. The utility of these new agents may be validated by the high proportion of Enterobacterales causing UTIs [8, 9], which corresponds well with our current analysis of contemporary data from the Premier Healthcare Database. In this analysis, among UTIs caused by CR pathogens, *P. aeruginosa* was the most frequent pathogen followed by *K. pneumoniae*, and a small proportion were caused by *S. maltophilia* and *A. baumannii*, suggesting that non-fermenter species causing UTIs frequently display carbapenem resistance. Overall, the proportion of CR pathogens was much greater among non-fermenter species than in Enterobacterales, representing a valid concern for physicians when selecting empiric antibiotics.

There was an apparent difference between male and female patients in the propensity to be infected with either CR or CS bacteria, reflecting the well-known difference in UTI aetiology. UTIs occur more frequently in females [1]. Female patients with uncomplicated infections, which are primarily caused by CS Enterobacterales and frequently recur, were included in the analysis. However, according to the data, hospitalised male patients with cUTIs more likely acquire CR infections due to the increased prevalence of these pathogens in hospitals. The higher proportion of CR UTIs among male patients was more predominant in the presence of bacteraemia.

The multivariable analysis confirmed that UTIs complicated by bacteraemia were more likely to be associated with male sex (OR 1.99, *P* < 0.0001). Male patients often present with complicated UTIs that frequently require hospitalisation, intravenous antibiotics, and instrumentation that can increase the risk of bacterial translocation into the bloodstream [1]. Other non-pathogen-related variables significantly associated with an increased likelihood of bacteraemic UTI were being non-white, age 45–65 years, ICU admission, undergoing urinary surgery or having a urinary catheter. The presence of such risk factors may lead to the deterioration of UTIs into sepsis [26, 27], indicating a poor prognosis. Complications, such as bacteraemia or sepsis, may require ICU admission with a prolonged hospital overall LOS and increased hospital charges, as suggested by our analysis. Patients with CR UTIs remained for significantly longer in the ICU compared with those with CS UTIs, particularly when UTI was complicated with bacteraemia. Management of CR infections complicated with bacteraemia or sepsis could be challenging with limited treatment options, particularly as the availability of agents with high activity against non-fermenters is limited [6, 8, 23].

Although, the overall risk of mortality in UTI patients is relatively low compared with other serious Gram-negative infections [9, 27], our analysis demonstrated that the proportion of patients who died in the hospital with bacteraemic CR infections (10.5%) was approximately twice that of bacteraemic CS (6.0%) or non-bacteraemic CR infections (4.0%), suggesting an added burden for such patients. A lack of, or failure to promptly initiate, appropriate antibiotic therapy increases the risk of mortality, particularly when identification of a CR pathogen is delayed [15, 28]. However, appropriate antibiotic treatment for CR UTIs may not be timely due to the delayed admission following index culture, leading to the possibility of a nosocomial origin of the CR pathogen. Therefore, understanding risk factors and predicting the presence of bacteraemia due to CR pathogens could almost be considered essential to enable timely administration of appropriate therapy and to reduce risk of mortality [15].

Among survivors, patients with CR infections were less likely to be discharged home (38.4% vs 51.0%) and more likely to be transferred to another facility (42.2% vs 33.0%). Furthermore, a large proportion of patients with CR infections, among those who were alive at the time of discharge, were readmitted with the same pathogen (22.6%), nearly double the proportion of those with CS infections (13.5%). The more frequent transfer to a different unit and readmission may contribute to the escalated burden in terms of hospitalisation charges for CR vs CS infections.

The strengths of the current study were the use of a large database, consecutive patients’ data over 4–5 years, with species and carbapenem susceptibility information available. Only patients for whom susceptibility of the causative pathogen was known and the source of infection was confirmed as urine were included in the analysis. The analysed population represents a large, hospitalised patient population who required inpatient management, probably with intravenous antibiotic treatment.

Limitations of the study included specific analyses for the type of antibiotic treatment used prior to and during the hospitalisation and disease severity for these patients. The diagnosis of UTI was based on the presence of Gram-negative bacteria in urine samples in hospitalised patients receiving antibiotic treatment, which cannot rule out those who were only colonised. Because of these, the study could not exclude patients with acute uncomplicated pyelonephritis, which may have a different burden than cUTIs.

## Conclusions

Our analysis clearly demonstrates the increased burden associated with the presence of CR pathogens in patients with UTIs, particularly those with infections complicated by bacteraemia, in terms of the risk of overall as well as infection-associated LOS and ICU LOS, readmissions, healthcare charges and, in patients with bacteraemia, increased risk of mortality. Early identification of UTI patients at high risk of bacteraemia and/or CR pathogens is necessary to reduce this burden for hospitals and patients.

## Supplementary Information

**Additional file 1: Supplementary Table 1.** Time to death, Day 14 and Day 30 mortality, and mortality by pathogen.

**Additional file 2: Supplementary Table 2.** Mortality rate by pathogen in overall UTI group, and with or without bacteraemia.

**Additional file 3: Supplementary Table 3.** LOS charges according to presence of bacteraemia and carbapenem resistance.

**Additional file 4: Supplementary Table 4.** Univariate analyses of baseline characteristics to assess the risk for urosepsis.

**Additional file 5: Supplementary Fig. 1.** Patient attrition from the Premier HealthCare Database between 2014 and 2019. *ICD* International Classification of Diseases, *UTI* Urinary tract infection. ^a^This population excludes patients with cultures taken prior to admission (*n* = 64). This is to avoid the situation of missing data between the index culture date and the admission date. ^b^This population excludes the 30,746 patients who were not part of the ‘UTI with bacteraemia’ population but who had an ICD diagnosis of sepsis and/or evidence of positive blood culture at any time.

## Data Availability

The data that support the findings of this study are available from Premier Inc., but restrictions apply to the availability of these data, which were used under license for the current study, and so are not publicly available. Data are however available from the authors upon reasonable request and with permission of Premier Inc.

## Abbreviations

CA: Community acquired
CCI: Charlson Comorbidity Index
CR: Carbapenem-resistant
CRE: Carbapenem-resistant Enterobacterales
CS: Carbapenem-susceptible
cUTI: Complicated urinary tract infection
HA: Hospital acquired
HAI: Hospital-acquired infection
HIV: Human immunodeficiency virus
ICU: Intensive care unit
LOS: Length of stay
OR: Odds ratio
SD: Standard deviation
SNF: Skilled nursing facility
UTI: Urinary tract infection
