# Supplementary material for: Burden of illness in US hospitals due to carbapenem-resistant Gram-negative urinary tract infections in patients with or without bacteraemia
Source: BMC Infect Dis. 2021 Jun 14;21:572. doi: 10.1186/s12879-021-06229-x (PMC8201721; doi:10.1186/s12879-021-06229-x)
Supplement: Supplementary file 1 — Additional file 1: Supplementary Table 1. Time to death, Day 14 and Day 30 mortality, and mortality by pathogen. [file 12879_2021_6229_MOESM1_ESM.docx]

**Supplementary Table 1** Time to death, Day 14 and Day 30 mortality, and mortality by pathogen

| **Characteristic** | **Overall, N=47,496** | | | | **With bacteraemia, n=11,629** | | | **Without bacteraemia, n=35,867** | | |
| --- | --- | --- | --- | --- | --- | --- | --- | --- | --- | --- |
|  | **CR, n=2076** | | **CS, n=45,420** | ***P* value** | **CR, n=201** | **CS, n=11,428** | ***P* value** | **CR, n=1875** | **CS, n=33,992** | ***P* value** |
| **Number of in-hospital deaths** | n=96 | | n=2100 |  | n=21 | n=690 |  | n=75 | n=1410 |  |
| Inpatient mortality within 14 and 30 days of index urine culture, n (%) | |  |  |  |  |  |  |  |  |  |
| Death within 14 days | 70 (3.4) | | 1784 (3.9) | 0.201 | 12 (6.0) | 586 (5.1) | 0.592 | 58 (3.1) | 1198 (3.5) | 0.323 |
| Death within 30 days | 86 (4.1) | | 2025 (4.5) | 0.495 | 16 (8.0) | 665 (5.8) | 0.200 | 70 (3.7) | 1360 (4.0) | 0.564 |

*CR* Carbapenem resistant, *CS* Carbapenem susceptible, *SD* standard deviation
