# Supplementary material for: Burden of illness in US hospitals due to carbapenem-resistant Gram-negative urinary tract infections in patients with or without bacteraemia
Source: BMC Infect Dis. 2021 Jun 14;21:572. doi: 10.1186/s12879-021-06229-x (PMC8201721; doi:10.1186/s12879-021-06229-x)
Supplement: Supplementary file 2 — Additional file 2: Supplementary Table 2. Mortality rate by pathogen in overall UTI group, and with or without bacteraemia. [file 12879_2021_6229_MOESM2_ESM.docx]

**Supplementary Table 2** Mortality rate by pathogen in overall UTI group, and with or without bacteraemia

|  | Mortality, n/N (%) | | | | | |
| --- | --- | --- | --- | --- | --- | --- |
| Pathogen in the index urine sample | **Overall, N=47,496** | | **With bacteraemia, n=11,629** | | **Without bacteraemia, n=35,867** | |
|  | **CR, n=2076** | **CS, n=45,420** | **CR, n=201** | **CS, n=11,428** | **CR, n=1875** | **CS, n=33,992** |
| Number of in-hospital deaths, n (%) | n=96 | n=2100 | n=21 | n=690 | n=75 | n=1410 |
| *A. baumannii* | 4/95 (4.2) | 1/80 (1.3) | 1/8 (12.5) | 0/9 (0) | 3/87 (3.4) | 1/71 (1.4) |
| *E. aerogenes* | 1/42 (2.4) | 30/619 (4.8) | 0/7 (0) | 5/134 (3.7) | 1/35 (2.9) | 25/485 (5.2) |
| *E. coli* | 4/80 (5.0) | 1223/29,311 (4.2) | 2/10 (20.0) | 421/8106 (5.2) | 2/70 (2.9) | 802/21,205 (3.8) |
| *E. cloacae* | 10/153 (6.5) | 66/1202 (5.5) | 2/15 (13.3) | 14/172 (8.1) | 8/138 (5.8) | 52/1030 (5.0) |
| *K. oxytoca* | 0/4 (0) | 46/899 (5.1) | NA | 11/121 (9.1) | 0/4 (0) | 35/778 (4.5) |
| *K. pneumoniae* | 12/294 (4.1) | 410/7403 (5.5) | 3/45 (6.7) | 114/1545 (7.4) | 9/249 (3.6) | 296/5858 (5.1) |
| *M. morganii* | 3/58 (5.2) | 28/544 (5.1) | 0/2 (0) | 6/61 (9.8) | 3/56 (5.4) | 22/483 (4.6) |
| *P. aeruginosa* | 49/1026 (4.8) | 137/3069 (4.5) | 11/88 (12.5) | 31/311 (10.0) | 38/938 (4.1) | 106/2758 (3.8) |
| *P. mirabilis* | 3/111 (2.7) | 229/3948 (5.8) | 1/16 (6.3) | 88/945 (9.3) | 2/95 (2.1) | 141/3003 (4.7) |
| *S. marcescens* | 1/6 (16.7) | 24/425 (5.6) | NA | 9/121 (7.4) | 1/6 (16.7) | 15/304 (4.9) |
| *S. maltophilia* | 10/235 (4.3) | NA | 1/10 (10.0) | NA | 9/225 (4.0) | NA |

*CR* Carbapenem resistant, *CS* Carbapenem susceptible, *NA* Not available
