# Supplementary material for: Burden of illness in US hospitals due to carbapenem-resistant Gram-negative urinary tract infections in patients with or without bacteraemia
Source: BMC Infect Dis. 2021 Jun 14;21:572. doi: 10.1186/s12879-021-06229-x (PMC8201721; doi:10.1186/s12879-021-06229-x)
Supplement: Supplementary file 3 — Additional file 3: Supplementary Table 3. LOS charges according to presence of bacteraemia and carbapenem resistance. [file 12879_2021_6229_MOESM3_ESM.docx]

**Supplementary Table 3** LOS charges according to presence of bacteraemia and carbapenem resistance

| **Characteristic** | **Overall, N=47,496** | | | **With bacteraemia, n=11,629** | | | **Without bacteraemia, n=35,867** | | |
| --- | --- | --- | --- | --- | --- | --- | --- | --- | --- |
|  | **CR, n=2076** | **CS, n=45,420** | ***P* value** | **CR, n=201** | **CS, n=11,428** | ***P* value** | **CR, n=1875** | **CS, n=33,992** | ***P* value** |
| Total LOS charges^a^ (US$) |  |  |  |  |  |  |  |  |  |
| *Mean (SD)* | 91,752 (245,191) | 66,011 (121,934) | <0.001 | 178,176 (383,931) | 64,838 (104,556) | <0.001 | 82,488 (223,491) | 66,405 (127,244) | 0.002 |
| *Median* | 44,603 | 36,957 | <0.001 | 75,070 | 38,930 | <0.001 | 42,797 | 36,223 | <0.001 |
| *Q1–Q3* | 25,611.1–83,155.3 | 21,500.3–69,490.3 |  | 36,362.5–133,010.0 | 23,446.3–69,079.6 |  | 24,548.6–78,085.0 | 20,858.6–69,717.6 |  |
| Total ICU LOS charges^b^ (US$) |  |  |  |  |  |  |  |  |  |
| *Mean (SD)* | 28,792 (61,287) | 20,243 (36,399) | <0.001 | 48,798 (97,535) | 18,758 (33,822) | 0.001 | 24,690 (49,917) | 20,966 (37,572) | 0.084 |
| *Median* | 10,245 | 8894 | 0.226 | 11,904 | 9400 | 0.009 | 9450 | 8673 | 0.610 |
| *Q1–Q3* | 2282.0–25,068.0 | 2963.0–22,256.1 |  | 4430.0–36,297.0 | 4104.0–19,974.0 |  | 1945.5–23,859.5 | 2125.0–23,650.0 |  |
| Total infection-associated ICU LOS charges^c^ (US$) |  |  |  |  |  |  |  |  |  |
| *Mean (SD)* | 19,761 (34,104) | 17,393 (28,495) | 0.119 | 29,596 (46,607) | 17,815 (28,828) | 0.013 | 17,419 (29,980) | 17,151 (28,302) | 0.850 |
| *Median* | 10,168 | 8920 | 0.287 | 13,077 | 9580 | 0.016 | 9597 | 8446 | 0.505 |
| *Q1–Q3* | 3449.0–20,823.5 | 3635.0–19,656.0 |  | 4689.5–31,657.5 | 4447.0–19,557.0 |  | 3134.0–18,243.5 | 2862.0–19,692.0 |  |

*CR* Carbapenem resistant, *CS* Carbapenem susceptible, *ICU* Intensive care unit, *LOS* Length of stay, *SD* Standard deviation, *UTI* Urinary tract infection

^a^LOS-associated charge: the amount related to the hospitalisation duration that the hospital charged to the insurance companies.

^b^ICU LOS charge: the amount related to ICU stay length that the hospital charged to the insurance companies.

^c^Infection-associated ICU LOS charge: the amount related to the Infection-associated ICU stay length that the hospital charged to the insurance companies.
