# Supplementary material for: Burden of illness in US hospitals due to carbapenem-resistant Gram-negative urinary tract infections in patients with or without bacteraemia
Source: BMC Infect Dis. 2021 Jun 14;21:572. doi: 10.1186/s12879-021-06229-x (PMC8201721; doi:10.1186/s12879-021-06229-x)
Supplement: Supplementary file 4 — Additional file 4: Supplementary Table 4. Univariate analyses of baseline characteristics to assess the risk for urosepsis. [file 12879_2021_6229_MOESM4_ESM.docx]

**Supplementary Table 4** Univariate analyses of baseline characteristics to assess the risk for urosepsis

| Characteristics | Urosepsis | UTI only | *P* value |
| --- | --- | --- | --- |
|  | **n=11,629** | **n=35,867** | **(Chi-square)** |
| Age Group, n (%) |  |  |  |
| *18–45* | 1076 (9.3) | 3759 (10.5) | <0.0001 |
| *46–65* | 3279 (28.2) | 8877 (24.7) |  |
| *>65* | 7274 (62.6) | 23,231 (64.8) |  |
| Gender, n (%) |  |  |  |
| *Female* | 6756 (58.1) | 24,691 (68.8) | <0.0001 |
| *Male* | 4873 (41.9) | 11,176 (31.2) |  |
| Race, n (%) |  |  |  |
| *Non-white* | 3284 (28.2) | 8754 (24.4) | <0.0001 |
| *White* | 8345 (71.8) | 27,113 (75.6) |  |
| Admitted to ICU, n (%) |  |  |  |
| *Yes* | 3663 (31.5) | 6624 (18.5) | <0.0001 |
| *No* | 7966 (68.5) | 29,243 (81.5) |  |
| Had urine catheters, n (%) |  |  |  |
| *Yes* | 3031 (26.1) | 7170 (20.0) | <0.0001 |
| *No* | 8598 (73.9) | 28,697 (80.0) |  |
| Had urinary surgery, n (%) |  |  |  |
| *Yes* | 2136 (18.4) | 4352 (12.1) | <0.0001 |
| *No* | 9357 (80.5) | 31,149 (86.8) |  |
| Pathogens, n (%) |  |  |  |
| *A. baumannii* | 16 (0.1) | 135 (0.4) | <0.0001 |
| *P. aeruginosa* | 394 (3.4) | 3255 (9.1) |  |
| *S. maltophilia* | 10 (0.1) | 216 (0.6) |  |
| *E. coli* | 8045 (69.2) | 19,890 (55.5) |  |
| *K. pneumoniae* | 1557 (13.4) | 5217 (14.5) |  |
| *Other* | 1607 (13.8) | 7154 (19.9) |  |
| CR status of the pathogen, n (%) |  |  |  |
| *CR* | 201 (1.7) | 1875 (5.2) | <0.0001 |
| *CS* | 11,428 (98.3) | 33,992 (94.8) |  |
| Onset, n (%) |  |  |  |
| *HAI* | 531 (4.6) | 5617 (15.7) | <0.0001 |
| *Community onset* | 11,098 (95.4) | 30,250 (84.3) |  |
| Admission source, n (%) |  |  |  |
| *Clinic* | 808 (6.9) | 2972 (8.3) |  |
| *Non-healthcare facility point of origin* | 9269 (79.7) | 26,962 (75.2) |  |
| *Transferred* | 1191 (10.2) | 5043 (14.1) |  |
| *Information not available* | 361 (3.1) | 890 (2.5) |  |
| Admission type, n (%) |  |  |  |
| *Elective* | 392 (3.4) | 2606 (7.3) | <0.0001 |
| *Emergency* | 10,399 (89.4) | 29,492 (82.2) |  |
| *Information not available* | 18 (0.2) | 103 (0.3) |  |
| *Trauma centre* | 17 (0.1) | 182 (0.5) |  |
| *Urgent* | 803 (6.9) | 3484 (9.7) |  |
| CCI, n (%) |  |  |  |
| *0* | 2077 (17.9) | 5369 (15.0) | <0.0001 |
| *1* | 2307 (19.8) | 6174 (17.2) |  |
| *2* | 2050 (17.6) | 6473 (18.0) |  |
| *3–5* | 3724 (32.0) | 12,383 (34.5) |  |
| *5+* | 1471 (12.6) | 5468 (15.2) |  |
| Day from admission to index culture, n (%) |  |  |  |
| *Same day* | 10,827 (93.1) | 26,871 (74.9) | <0.0001 |
| *2^nd^–4^th^ day* | 362 (3.1) | 5204 (14.5) |  |
| *5^th+^ days* | 440 (3.8) | 3792 (10.6) |  |
| Mono- vs polymicrobial, n (%) |  |  |  |
| *Monomicrobial* | 11,534 (99.2) | 33,899 (94.5) | <0.0001 |
| *Polymicrobial* | 95 (0.8) | 1968 (5.5) |  |

*CCI* Charlson Comorbidity Index, *CR* Carbapenem resistant, *CS* Carbapenem susceptible, *HAI* Hospital-acquired infection (i.e. after 3 days), *ICU* Intensive care unit, *UTI* Urinary tract infection
