# Supplementary material for: Burden of illness in US hospitals due to carbapenem-resistant Gram-negative urinary tract infections in patients with or without bacteraemia
Source: BMC Infect Dis. 2021 Jun 14;21:572. doi: 10.1186/s12879-021-06229-x (PMC8201721; doi:10.1186/s12879-021-06229-x)
Supplement: Supplementary file 5 — Additional file 5: Supplementary Fig. 1. Patient attrition from the Premier HealthCare Database between 2014 and 2019. ICD International Classification of Diseases, UTI Urinary tract infection. aThis population excludes patients with cultures taken prior to admission (n = 64). This is to avoid the situation of missing data between the index culture date and the admission date. bThis population excludes the 30,746 patients who were not part of the ‘UTI with bacteraemia’ population but who had an ICD diagnosis of sepsis and/or evidence of positive blood culture at any time. [file 12879_2021_6229_MOESM5_ESM.docx]

**Supplementary Fig. 1** Patient attrition from the Premier HealthCare Database between 2014 and 2019


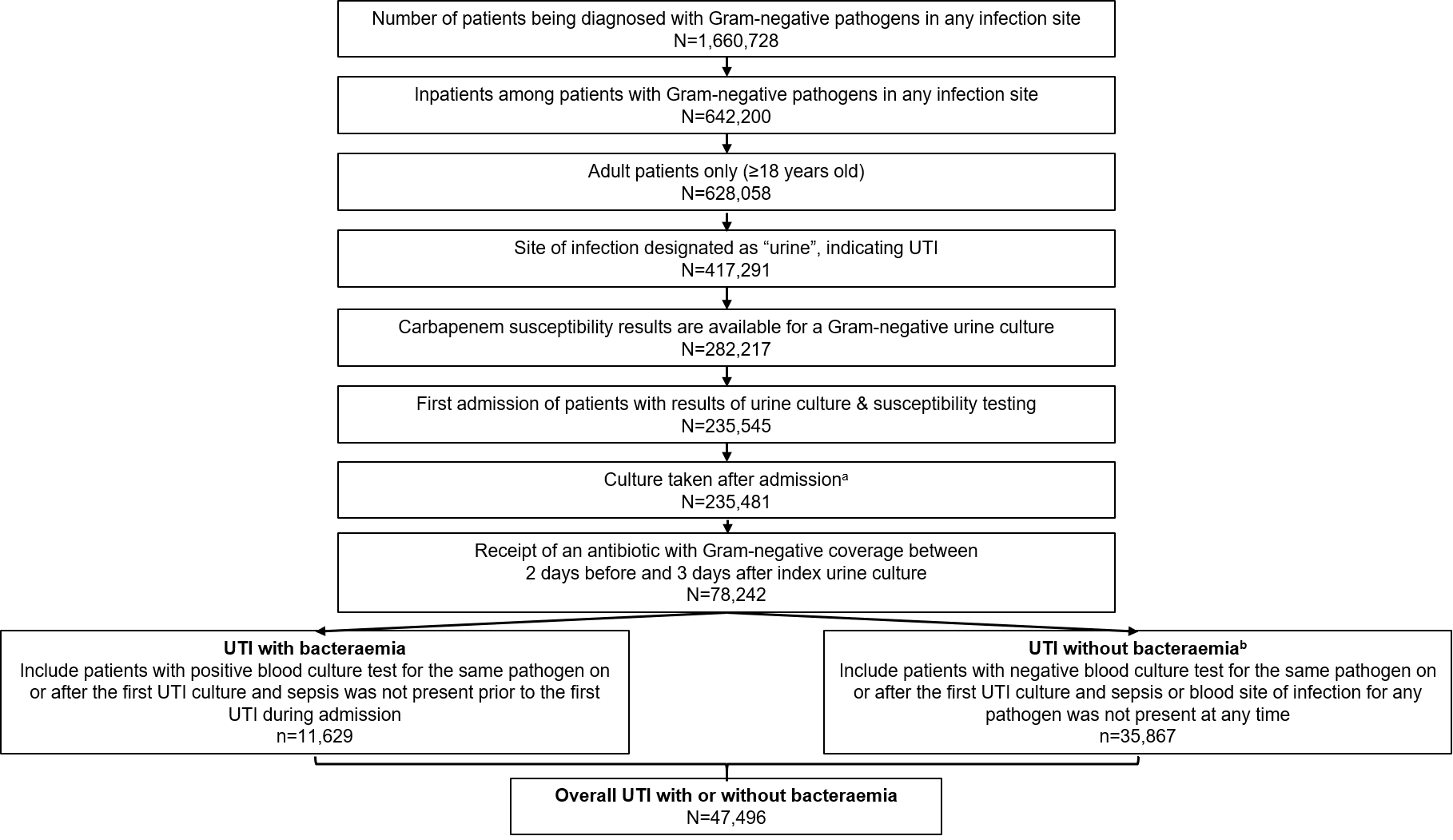


*ICD* International Classification of Diseases, *UTI* Urinary tract infection

^a^This population excludes patients with cultures taken prior to admission (n=64). This is to avoid the situation of missing data between the index culture date and the admission date.

^b^This population excludes the 30,746 patients who were not part of the ‘UTI with bacteraemia’ population but who had an ICD diagnosis of sepsis and/or evidence of positive blood culture at any time.
